# Coherence of Stroke Survivors’ Lived Experiences and the Stroke-Specific Quality of Life Scale

**DOI:** 10.1001/jamanetworkopen.2025.37951

**Published:** 2025-10-17

**Authors:** Devanshi Choksi, Mary Craven, Taylor McVeigh, Akashleena Mallick, Tanzeela Ranman, Christina Kourkoulis, Sofia Constantinescu, Rachel Kitagawa, Emilie Egger, Lindsay Rosenfeld, Rachel Forman, Guido J. Falcone, Jonathan Rosand, Vanessa L. Merker, Nirupama Yechoor

**Affiliations:** 1 Department of Neurology, Massachusetts General Hospital, Boston; 2 Brain Care Labs, Massachusetts General Hospital, Boston; 3 Department of Neurology, Yale School of Medicine, New Haven, Connecticut; 4 Department of Psychiatry, Perelman School of Medicine, Philadelphia, Pennsylvania; 5 Institute for Child, Youth, and Family Policy, Heller School for Social Policy and Management, Brandeis University, Waltham, Massachusetts; 6 Department of Social and Behavioral Sciences, Harvard T.H. Chan School of Public Health, Boston, Massachusetts; 7 Division of Neurocritical Care, Massachusetts General Hospital, Boston

## Abstract

**Importance:**

Physical, behavioral, and social outcomes after stroke are determined by the complex interaction of external states (eg, mobility) and internal states (eg, emotional distress). Improving these outcomes, collectively called health-related quality of life (HRQOL), requires their accurate measurement.

**Objectives:**

To identify important HRQOL constructs that are associated with poststroke recovery and assess whether the Stroke-Specific Quality of Life (SSQOL) Scale, which measures 12 physical and psychosocial outcomes, adequately captures HRQOL for survivors of stroke.

**Design, Setting, and Participants:**

This qualitative study was conducted between October 1, 2023, and December 31, 2024, at 2 US comprehensive stroke centers. Stroke survivors and caregivers participated in 2 consecutive focus group sessions: the first on physical well-being and the second on emotional well-being. All stroke survivors completed the SSQOL survey.

**Exposures:**

Physical and emotional well-being in recovery after stroke.

**Main Outcomes and Measures:**

Identification of themes regarding HRQOL constructs and their coherence with the SSQOL Scale. The framework method was used for qualitative analysis, and joint displays were used to assess coherence between qualitative themes and SSQOL Scale domains.

**Results:**

A total of 41 participants (median age, 55 years [range, 27-77 years]; 22 women [54%]) were recruited nationally; the cohort comprised 30 stroke survivors and 11 caregivers. Qualitative analysis demonstrated 5 key HRQOL constructs crucial to physical and emotional well-being in recovery. Reduced physical functioning and reduced social participation were key external states associated with recovery. Loss of independence, feelings of shame, and the fear of uncertainty were key internal states associated with HRQOL. This analysis confirms that the SSQOL Scale adequately captures the external states associated with HRQOL (reduced physical functioning and reduced social participation). However, internal states were only partially captured by SSQOL Scale.

**Conclusion and Relevance:**

This qualitative study of lived experiences among stroke survivors and caregivers suggested that internal states shape recovery but were not fully captured in a validated, widely used HRQOL assessment, highlighting a potential gap that may be unaddressed in routine poststroke care. Future studies are required to understand which combination of validated assessments may provide more comprehensive assessments of HRQOL during poststroke recovery.

## Key Points

**Question:**

Does the Stroke-Specific Quality of Life (SSQOL) Scale capture the lived experiences of stroke survivors during recovery?

**Findings:**

This qualitative study including 41 participants identified 5 health-related quality of life constructs that are associated with recovery: loss of independence, decreased physical mobility, feelings of shame, fear of uncertainty, and reduced community participation. The SSQOL Scale captured decreased physical mobility and reduced community participation but did not capture the constructs of loss of independence, feelings of shame, and fear of uncertainty.

**Meaning:**

This study suggests that relying solely on the SSQOL Scale to assess patient-centered outcomes may miss important experiences that are associated with quality of life in stroke recovery.

## Teaser

This qualitative study examines health-related quality of life constructs associated with poststroke recovery and assesses whether the Stroke-Specific Quality of Life (SSQOL) Scale adequately captures health-related quality of life for survivors of stroke.

## Introduction

Stroke recovery involves the complex interaction of physical, cognitive, and psychosocial states that shape how individuals adapt to life after stroke. Health-related quality of life (HRQOL) constructs, defined as an individual’s perceptions of physical, mental, and social health,^1^ are increasingly recognized as important outcomes in stroke recovery research.^2,3,4^ Along with physical impairments, stroke survivors face altered self-identity and loss of independence as they adapt to the changes in their relationships and daily activities.^5,6,7^ These role changes further influence overall health, well-being, and medical care,^8^ highlighting the importance of understanding the HRQOL constructs in stroke recovery.^9^

Quantitative measures of HRQOL have been developed, including the Stroke-Specific Quality of Life (SSQOL) Scale,^2^ which is a disease-specific survey designed to evaluate 12 domains of physical and psychosocial functioning.^10,11^ Although the SSQOL Scale was developed with input from stroke survivors, it is restricted by prespecified domains and known floor and ceiling effects,^12^ limiting the ability to capture subtle changes during recovery. In addition, even reliable measures can incorrectly estimate change due to unaddressed HRQOL constructs, called *missing concepts*.^13^ Thus, it is unclear if the SSQOL Scale adequately captures the nuanced experiences of stroke survivors.

To support person-centered outcome measurements, it is important to identify HRQOL constructs that align with stroke survivors’ lived experiences. Qualitative research enables researchers to assess the coherence between commonly used standardized assessments and lived experiences. The primary objective of this study was to identify key HRQOL constructs and measure the coherence between the SSQOL Scale and stroke survivors’ lived experiences in recovery.

## Methods

### Study Setting and Design

A prospective qualitative study was conducted from October 1, 2023, until December 31, 2024, at 2 quaternary-care academic medical centers that are certified as comprehensive stroke centers.^14^ A total of 6 focus groups, each comprising 5 to 8 participants, were conducted at the 2 sites. Each focus group met for 2 consecutive sessions, with the first session focusing on physical well-being and the second session focusing on emotional well-being in stroke recovery. All focus groups were approximately 60 minutes and were conducted over secure, Health Insurance Portability and Accountability Act–protected online Zoom conferences.^15^ The SSQOL Scale was administered at the end of the first session. Participants were compensated for their time and contributions to the study: they were given $30 (USD) per session attended, with a maximum amount of $210. Ethical approval was obtained from the Massachusetts General Hospital and Yale School of Medicine institutional review boards. All participants provided voluntary written informed consent to study participation and completed both focus group sessions. To maintain confidentiality, survey responses were anonymized, and all transcripts were deidentified and uploaded to a secure cloud platform. The Consolidated Criteria for Reporting Qualitative Research (COREQ) reporting guideline was followed to ensure transparent reporting of the methodology.

### Participant Recruitment and Demographics

Nationwide recruitment of study participants was conducted through online platforms. Other recruitment methods included posting the fliers on social media platforms and through community-based organizations. Purposive sampling was used to ensure a diverse range of representation by demographics. Inclusion criteria were (1) age 18 years or older, (2) history of stroke or serve as the primary caregiver for a person with history of stroke, (3) ability to read and speak English, and (4) available internet access. Consenting participants completed a demographics questionnaire (eTable 1 in Supplement 1) including participant race and ethnicity (Asian, Black [African American, West Indian, Afro-Caribbean or Afro-Latino or sub-Saharan African], Hispanic or Latino, White, or another race [American Indian or Alaska Native, Native Hawaiian or Other Pacific Islander, or other unspecified race or ethnicity] and/or >1 race or ethnicity). Because racial and ethnic disparities and inequalities in both acute treatment for stroke and poststroke outcomes have been described, we felt it was important to collect racial and ethnicity data to contextualize different lived experiences based on this prior work.

### Qualitative Data Collection and Analysis

Participants were informed that the goal of the study was to examine stroke recovery by understanding well-being. Focus groups at site 1 were conducted by a female neurointensivist with clinical expertise in stroke management (N.Y.). At site 2, focus groups were conducted by a female vascular neurologist (R.F.), a male neurointensivist (G.J.F.), and a female postgraduate clinical research associate (S.C). Although most participants had no prior relationship with the interviewers, some participants had received clinical care from the interviewers prior to the study.

A common interview guide was constructed with expertise from 4 investigators (E.E., R.F., G.J.F, and N.Y). The interview guide was used at both sites to facilitate focus group discussions, ensuring consistency in data collection across the 2 centers (eTable 2 in Supplement 1).

Data analysis was conducted using the framework method.^16^ Three independent coders at site 1 (D.C., A.M., and N.Y.) and site 2 (S.C., R.K., and E.E.) reviewed the transcripts and conducted open coding to develop a preliminary site-specific codebook. Researchers across both sites held regular meetings to triangulate the codes and develop a unified codebook, enhancing the credibility of analysis.^17^ For example, codes such as “identity” or “isolation” were grouped under a broader parent code labeled “emotional impact” that defined association with emotional well-being. Transcripts were recoded using unified codebooks to ensure validity and consistency. Transcripts were not returned to the participants for corrections and no repeat interviews were carried out.

### Quantitative Data Collection and Analysis

The SSQOL surveys were administered to stroke survivors using RedCap^18^ at site 1 and via telephone at site 2. Participants rated each item on the SSQOL Scale using a 5-point Likert scale, with a score of 1 corresponding to “strongly agree” and a score of 5 corresponding to “strongly disagree.”

### Examining Coherence Between Qualitative Data and SSQOL Scale Domains

To ensure methodological rigor, 2 investigators (D.C. and N.Y.) met regularly to assess coherence between qualitative and quantitative data using an iterative and reflexive approach. Each qualitative theme was compared with prespecified and relevant SSQOL Scale domains to examine coherence based on the extent to which the SSQOL Scale domains captured participant perspectives. The findings demonstrated confirmation when the survey domains captured identified themes. If the themes did not align with the SSQOL Scale domains, then they were determined to be discordant.^19,20^

## Results

### Participant Characteristics

The mean (SD) value for each domain on the SSQOL Scale is reported to characterize the sample (Table 1). A total of 41 individuals (median age, 55 years [range, 27-77 years]; 22 women [54%] and 19 men [46%]; 3 Asian participants [7%], 7 Black participants [17%], 4 of 40 Hispanic or Latino participants [10%]; 28 White participants [68%], and 3 participants of another race and/or >1 race or ethnicity [7%]) participated in our study (Table 2). The cohort comprised 30 stroke survivors and 11 caregivers. The sample included participants with a range of self-reported modified Rankin Scale scores; 73% of participants (29 of 40) reported no significant to slight disability. Adverse Childhood Experience scores^21,22,23^ and self-reported general well-being status (assessed via the 36-item Short-Form Survey),^24,25^ both of which have been associated with overall well-being, are reported in Table 2.

**Table 1.** Values for the SSQOL Scale Domains (N = 27)

SSQOL scale domain	Value, mean (SD)^a^
Energy	3.30 (1.20)
Family	3.59 (1.19)
Language	4.30 (0.85)
Mobility	3.72 (1.34)
Mood	3.94 (0.98)
Personality	3.52 (1.04)
Self-care	4.41 (0.99)
Social roles	3.06 (1.35)
Thinking	3.41 (1.19)
Upper extremity function	4.12 (1.01)
Vision	4.65 (0.69)
Work or productivity	3.25 (1.15)

Abbreviation: SSQOL, Stroke-Specific Quality of Life.

^a^ The values for all domains have a range of 1 to 5.

**Table 2.** Participant Characteristics

Participant characteristic	Value
Total focus groups, No.	6
Total participants, No.	41
Stroke survivors, No.	30
Caregivers, No.	11
Gender, No./total No. (%)	
Male	19/41 (46)
Female	22/41 (54)
Age, median (range), y	55 (27-77)
Race, No./total No. (%)	
Asian	3/41 (7)
Black^a^	7/41 (17)
White	28/41 (68)
Some other race or >1 race^b^	3/41 (7)
Ethnicity, No./total No. (%)^c^	
Hispanic or Latino	4/40 (10)
Non-Hispanic or Latino	36/40 (90)
Income, No./total No. (%), $^d^	
<20 000	3/34 (7)
20 000-34 999	5/34 (12)
35 000-49 999	1/34 (2)
50 000-74 999	4/34 (10)
75 000-99 999	4/34 (10)
≥100 000	17/34 (42)
Modified Rankin Scale score, median (range)^e^	1 (1-5)
36-Item Short-Form Survey score, No./total No. (%)^f^	
Excellent	2/41 (5)
Fair	8/41 (20)
Good	13/41 (32)
Poor	2/41 (5)
Very good	11/41 (27)
Somewhat better than 1 year ago	2/41 (5)
About the same as 1 year ago	2/41 (5)
Much better now than 1 year ago	1/41 (3)
Adverse Childhood Experiences score, median (range)	1 (1-9)

^a^ African American, West Indian, Afro-Caribbean or Afro-Latino, or African (sub-Saharan).

^b^ Native Hawaiian or Other Pacific Islander or American Indian or Alaska Native, or other unspecified.

^c^ No. of missing observations = 1.

^d^ No. of missing observations = 7.

^e^ Scores range from 0 to 6, with 0 indicating no symptoms; 1, symptoms without clinical disability; 2, slight disability; 3, moderate disability; 4, moderately severe disability; 5, severe disability; and 6, death.

^f^ Participant’s health is based on a set of generic, coherent, and easily administered quality-of-life measures. These measures rely on patient self-reporting.

### Qualitative Themes

Qualitative analysis revealed 5 HRQOL constructs: loss of independence, decreased physical mobility, feelings of shame, the fear of uncertainty, and reduced community participation. Each construct is described in further detail and compared with SSQOL Scale domains (Table 3).

**Table 3.** Joint Display of Qualitative Themes Compared With SSQOL Scale Domains

Theme	Participant quote	SSQOL Scale Domain	Comparison with theme
Loss of independence	“I’m having difficulty, well I’m currently not driving, I’m in the passenger’s seat. But the lack of independence hits hard. I’m depending on others to do things for me, to bring me to appointments. That’s the hard part for me.”	Family roles; self-care; personality	Discordance: Although the items captured assistance required with daily tasks, impact of dependency, and behavioral changes, they did not fully align with loss of independence, marked by reduced autonomy, altered self-identity, and feelings of inadequacy.
Decreased physical mobility	“I couldn’t get in and out of the shower that had a tub just by lifting my legs over. They put a bench in there for me. But just getting around, physically, the mobility was not there.”	Mobility; energy; work or productivity	Confirmation: The items under these domains captured stroke survivors’ concerns regarding mobility, balance, and increased fatigue.
Feelings of shame	“I can’t go out tonight because I don’t know where the closest bathroom is, and I would stay at home because I was so afraid of not being able to get to a bathroom that I could actually get off the seat. And it was terrifying.”	Mood	Discordance: Although the items captured social isolation and discouragement, which can be a consequence of shame, none of the items specifically addressed shame as a construct.
Fear of uncertainty	“It [stroke] created a lot of fear for me. More fear, more fear than I had felt.”	Mood	Discordance: The items under the mood domain captured discouragement, which may partially align with concerns regarding the future. However, none of the items captured the heightened sense of anxiety regarding future capabilities.
Reduced community participation	“There’s a social aspect to the gym because you have your friends there. The people you see all the time or the people you take classes with, and I miss that interaction.”	Social roles	Confirmation: The items under social roles addressed participants’ concerns regarding social interactions and engaging in recreational activities.

Abbreviation: SSQOL, Stroke-Specific Quality of Life.

#### Loss of Independence

A loss of independence was noted primarily with daily tasks, including walking, showering, and driving, all of which were significantly associated with HRQOL.

I wasn’t able to drive. I ended up hiring a woman to drive me around. Until I bought a car retrofit[ted] for left-handed driv[ing], and then I had some independence, which cannot be overstated. When you lose your independence, you lose a lot mentally because you’re so dependent upon someone else to help you do things that you always took for granted… and once you get it back, you realize how much you don’t want to give it up again. (Participant 3, site 2)

I was very active—I did Zumba the day I had my stroke—and understanding that I couldn’t even walk 3 houses down without being shaky and coming home and needing a nap. And leaving the house as one person and coming back as somebody else. That was the hardest thing for me. (Participant 8, site 2)

Participants shared that poststroke reliance on caregivers to complete activities disrupted their sense of prestroke normalcy and identity, demonstrating that the loss of independence is a deterrent to HRQOL. SSQOL Scale domains that aligned with this theme included self-care, family roles, and personality. Although these domains examined functional aspects of daily activities, they failed to capture the psychological association of decreased autonomy and thus were classified as discordant (Table 3).

#### Decreased Physical Mobility

Decreased physical mobility was another deterrent to HRQOL. Participants reported that reductions in mobility led to increased activity completion time, increased fatigue, decreased physical safety, and limited autonomy.

They had to install a handicapped rail and the shower. I wasn’t able to get in and out of the tub… I got the medical seat, obviously everything took longer. But just getting around, the physically mobility was not there. (Participant 16, site 1)

When I was growing up, even as an adult, napping was something that just was not in my DNA. And I’ve taken several naps in a day in the last 6 months. I definitely get very tired. That’s just my body now. (Participant 15, site 2)

The SSQOL Scale domains assessing mobility, energy, and work and productivity demonstrated confirmation because they adequately captured the functional impairments expressed by participants (Table 3).

#### Feelings of Shame

Shame was an internal state that participants expressed in many different forms, including the shame of visible signs of disability and sense of personal identity. First, participants shared a reluctance to use assistive devices because they are seen as a visible sign of disability.

I had a walker and that actually helped me out a lot. Even though I was embarrassed to use it out in public because I was 22. (Participant 2, site 1)

The devices also served as a reminder of physical limitations and associated feelings of reduced self-efficacy and altered self-perception, leading to feelings of shame.

Outside of the house is different. I feel like people hold doors for me or do things differently. Because even though I’m happy that they do that, I’m not happy that they do it for me. I’m the disabled one now; I don’t like that. (Participant 20, site 1)

In contrast, participants with less physical disability shared that they faced skepticism from others because their disability was not apparent. Exposure to these judgments or anticipated discrimination caused feelings of shame.

We’re using a handicapped tag and people are commenting on how slow I’m walking in the grocery store or [asking] why do you need that tag? There’s all these emotional pieces to feeling like I have to justify that in my head. (Participant 16, site 1)

Caregivers also shared experiences where visitors would not recognize survivors’ challenges with aphasia because the disability was not visible. Many visitors unknowingly excluded survivors from social participation.

I think a lot of times in the beginning when people were coming to the hospital to visit with my stepmom, she was excited to have company. But the conversations would either go too fast… she’d lose words and people would finish her sentences for her. She knows the word and that was hard for her in the beginning. (Participant 3, site 1)

The SSQOL Scale mood domain partially aligned with social isolation and discouragement. No other items captured the emotional experiences of shame and the domain was therefore classified as discordant (Table 3).

#### Fear of Uncertainty

The fear of uncertainty was another recurrent HRQOL theme.

The trauma of having a stroke and not knowing what’s going on and not knowing what your abilities are was scary. (Participant 6, site 1)

Uncertainty surrounding health status, future capabilities, and stroke recurrence influenced participants’ emotional state and resulted in a heightened sense of anxiety regarding their health, and therefore was a fourth deterrent to HRQOL.

Before my stroke I had a little bit of anxiety for sure. I would overthink things, but nothing could compare to what happened after the stroke. Just a few weeks later, I had this migraine. I went to the hospital the next day because I was anxious that it was happening as a stroke… That was only the beginning of having that sense of fear of not knowing. (Participant 15, site 1)

The SSQOL Scale mood domain showed discordance because the items in this domain did not capture the heightened sense of anxiety (Table 3). Although the item related to discouragement aligns with survivors’ concerns regarding the future, the mood domain did not address fear among stroke survivors.

#### Reduced Community Participation

Finally, participants discussed how stroke was associated with the ability to maintain social connections or engage in personally valued activities, which led to increased isolation.

The thing that seemed to bother her the most was the fact that she couldn’t drive, so she couldn’t go have coffee with her friends. I think that was probably the hardest thing for her when she came home. (Participant 3, site 1)

I just won’t get up and go to knit night you know? As much as I used to enjoy that… There’s been carjackings and some robberies in our area. And you start to feel that anxiety. You’re always a target but am I more of a target now because of my deficits? (Participant 9, site 2)

The SSQOL Scale social roles domain was coherent with survivors’ community participation, because the items addressed key concerns regarding engaging in valued activities and maintaining social connections (Table 3).

### Data Integration

Mean (SD) SSQOL Scale scores for all participants are listed in Table 2 and demonstrate that the domains of energy, thinking, and work or productivity were the most affected. The Figure shows the frequency of codes from qualitative transcripts that generated the 5 major themes and compares their frequency with the quantitative survey domains. We found that the SSQOL Scale and self-reported modified Rankin Scale score dedicate multiple domains to external HRQOL and 1 internal HRQOL domain of loss of independence. The quantitative surveys either miss or dedicate only 1 domain to internal HRQOL constructs of fear of uncertainty and feelings of shame. To further examine how precisely survey domains measure HRQOL constructs, we created a joint display (Table 3). Our findings demonstrate that the SSQOL Scale effectively captured external states, such as decreased physical mobility and reduced community participation. However, the internal states of loss of independence, feelings of shame, and the fear of uncertainty were not fully captured by the SSQOL Scale. Given this discordance, when examining stroke recovery, the choice of assessments was associated with which outcomes can be measured.

**Figure.**
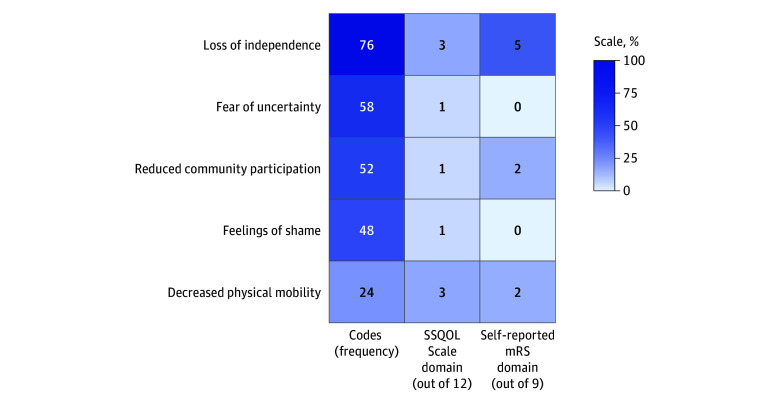
Frequency of Qualitative Codes by Theme Compared With Domains of Quantitative Surveys The figure demonstrates the frequency of themes in qualitative data and the number of domains of each quantitative survey that is dedicated to measuring components of the theme. Gradient color is represented by proportion out of 100; for example, the Stroke-Specific Quality of Life (SSQOL) Scale has 12 domains, and 3 domains are dedicated to measuring physical mobility and represent a proportion of 0.25. mRS indicates modified Rankin Scale.

## Discussion

In this qualitative study with stroke survivors and caregivers, we identified 5 key HRQOL constructs that were associated with poststroke recovery. In comparison with a commonly used HRQOL measure, we found that the SSQOL Scale effectively captured external states, such as reduced physical mobility and reduced community participation, but inadequately captured internal states, including loss of independence, feelings of shame, and the fear of uncertainty. The implications of our findings are 2-fold. The first implication is that stroke recovery encompasses more than physical recovery. The second implication is to highlight the importance of changes to internal states for stroke survivors, caregivers, and clinicians and to bring attention to their association with quality of life after stroke.

Similar to prior research, our findings highlight the association of losing independence with emotional well-being, through altered self-identity and lowered self-esteem.^5,26,27,28,29^ The self-discrepancy theory provides a useful framework to contextualize this finding^30^: stroke survivors’ dependence in routine tasks leads to a psychological conflict between their actual self (dependent on others) and their ideal self (prestroke independence).

Our analysis showed discordance between HRQOL internal states and SSQOL Scale domains. For example, the self-care domain queries functional loss of independence, but does not adequately capture the emotional challenges related to this loss. Similarly, the family roles domain queries the external association of dependency (eg, “I felt like a burden to my family”) but fails to capture the altered self-identity due to limited autonomy. Items on the personality domain measure behavioral changes but overlook feelings of inadequacy. These findings suggest that the SSQOL Scale may underestimate the psychological HRQOL constructs that shape stroke recovery.

Health-related stigma, where individuals face exclusion or anticipate adverse social judgment resulting in feelings of shame, emerged as an important HRQOL construct.^31,32^ The SSQOL Scale mood domain focuses on lack of interest, isolation, and discouragement, but does not capture experiences of shame. In other neurologic conditions such as multiple sclerosis, shame is a known barrier to seeking psychological help and is associated with reduced quality of life.^33,34^ Given that stroke survivors encounter shame, it is important to supplement the SSQOL Scale with additional measures, such as the Neuro-QoL Stigma Scale,^35^ for a comprehensive evaluation.

Similarly, the fear of uncertainty was associated with heightened emotional distress. However, the mood domain only captures anxiety about health status. Among individuals with chronic illness, uncertainty is negatively associated with self-efficacy, a key factor in disease management.^36,37^ Additional scales, such as the Mishel Uncertainty in Illness Scale,^38,39^ can complement the SSQOL Scale in capturing this construct.

The SSQOL Scale showed coherence with external states of HRQOL constructs. Reduced physical mobility confirmed overlap with mobility, energy, and work and productivity domains, indicating the SSQOL Scale’s comprehensiveness in capturing physical functioning, consistent with prior studies.^40,41^ Similarly, the social roles domain confirmed coherence with reduced community participation; both capture the impact of stroke survivors’ ability to maintain interpersonal relationships and participate in social activities.

### Strengths and Limitations

Our study has some strengths, including a prospective design that captures nuanced aspects of recovery that may not be captured in standardized assessments. In addition, conducting the study across 2 sites with national recruitment and a large cohort increases the generalizability of our findings. Our findings highlight HRQOL constructs that shape stroke recovery and may be missed in routine assessments but could potentially serve as important targets for functional recovery interventions.

Our study also has some limitations, including selection bias, given that participants may represent a highly motivated sample and virtual focus groups could exclude those with low digital literacy and low English language proficiency. Future studies should consider ensuring broader representation to include non-English speakers and those with limited digital literacy. Second, it is possible that variations in time since stroke may influence perceived HRQOL. Third, groups included both stroke survivors and caregivers to include those with severe disability. Future research is required to understand which HRQOL constructs are prioritized at different stages of recovery and conduct assessments in stroke survivors and caregivers separately.

## Conclusions

In this qualitative study of lived experiences with stroke survivors and caregivers, our findings suggest that the SSQOL Scale captures only the external HRQOL constructs, but it captures internal states less well. Our findings demonstrate that HRQOL components that shape recovery may be missed by widely used surveys, and futures studies are needed to determine which quantitative HRQOL measures effectively capture stroke survivors’ lived experiences.

## Notes

Supplement 1. **eTable 1.** Demographics Survey **eTable 2.** Interview Guide

Supplement 2.
Data Sharing Statement
