# Supplementary material for: Coherence of Stroke Survivors’ Lived Experiences and the Stroke-Specific Quality of Life Scale
Source: JAMA Netw Open. 2025 Oct 17;8(10):e2537951. doi: 10.1001/jamanetworkopen.2025.37951 (PMC12534846; doi:10.1001/jamanetworkopen.2025.37951)
Supplement: Supplement 1. — eTable 1. Demographics Survey eTable 2. Interview Guide [file jamanetwopen-e2537951-s001.pdf]

Supplementary Online Content

Choksi D, Craven M, McVeigh T, et al. Coherence of stroke survivors' lived experiences and the Stroke-Specific Quality of Life Scale. *JAMA Netw Open.* 2025;8(10):e2537951. doi:10.1001/jamanetworkopen.2025.37951

eTable 1. Demographics Survey

eTable 2. Interview Guide

This supplementary material has been provided by the authors to give readers additional information about their work.

© 2025 Choksi D et al. *JAMA Network Open.*

**eTable 1.** Demographics Survey
Full name
Age
Are you currently married, living with a partner, separated, divorced, widowed, or never married?<br>• Married<br>• Living with a partner<br>• Separated/Divorced<br>• Widowed<br>• Never married
If married, since what year?
Are you a stroke survivor or caregiver<br>• Caregiver<br>• Stroke survivor
What type of stroke did you/your loved one have?<br><br>Please provide any details you remember about the type of stroke.
How many people live in your household besides you?
How many are children? (Ages 0-18)
Who lives with you? Check all that apply.<br>• Spouse/Partner<br>• Child/Children/Stepchid<br>• Parents<br>• Grandchild/grandchildren<br>• Sibling(s)<br>• Aunt(s)/Uncle(s)<br>• Niece(s)/Nephew(s)<br>• Paid Aide/Housekeeper<br>• Friend(s) or roommate(s)<br>• Someone else<br>• I live alone
What is your zip code?
What is your gender identity?<br>• Female<br>• Male<br>• Non-binary<br>• Other<br>• Prefer not to answer
What is your total household income?<br>• 1 Less than $20,000<br>• 2 $20,000 to $34,999<br>• 3 $35,000 to $49,999<br>• 4 $50,000 to $74,999<br>• 5 $75,000 to $99,999<br>• 6 More than $100,000<br>• 7 Prefer not to answer
Ethnicity<br>• Hispanic or Latino

© 2025 Choksi D et al. *JAMA Network Open.*

• Not Hispanic or Latino
Race: Please check all racial categories you identify with.<br>• American Indian/Alaska Native<br>• African American, Black, West Indian, Afro-Caribbean, Afro-Latino, African (sub-Saharan)<br>• Asian- East or Southeast<br>• Asian- West or Southwest<br>• Asian- South<br>• Caucasian, White<br>• Native Hawaiian, Other Pacific Islander<br>• Some other race or origin
Education Level (select the highest level attained)<br>• Never attended/Kindergarten only<br>• 1st-8th grade<br>• 9th-12th grade (No diploma)<br>• High school graduate/GED<br>• Post high school vocational, technical, or trade school<br>• Some college but no degree<br>• Associate's degree<br>• Bachelor's degree<br>• Master's degree<br>• Professional or doctoral degree
Are you currently working or the primary caregiver for someone?<br>• Yes, Full Time (>30 hours/week)<br>• Yes, Part time (less than 30 hours/week)<br>• No<br>• Other (please specify)
Religion
Have you ever participated in a research study<br>• Yes<br>• No

© 2025 Choksi D et al. *JAMA Network Open.*

**eTable 2.** Interview Guide
**<u>Physical Wellbeing:</u>**
**Grand Tour Question 1: Physical capacity on discharge from the hospital**<br>**Clarification Questions**
• Did you go home? If not, where did you go?
• Did you have access to therapies? Did you feel like it was enough?
• What types of therapies did you need?
• How long did you stay in therapy services?
**Grand Tour Question 2: If and when you and your loved one returned home, what were the challenges to physical wellbeing?**
**Clarification Questions**
• What resources would have made the physical challenges you faced easier?
• What resources that were provided were not useful?
• Did you have difficulty with accessibility in the home?
**Grand Tour Question 3: If and when you and your loved one returned home, what helped in the transition back to your home?**<br>**Clarification Questions**
• What resources did you have access to that helped you physically?
• What resources did you find on your own that your doctor did not tell you about, if any?
**Grand Tour Question 4: What were the challenges outside of the home? In your neighborhood environment?**<br>**Clarification Questions**
• What were expected challenges in the community?
• What were unexpected resources that made life easier?
• What could change at the community level that could help stroke survivors?
**Grand Tour Question 5: Are doctor's offices equipped for people with new physical disabilities?**<br>**Clarification Questions**
• Did you need special licenses, such as a handicapped placard, after your stroke?
• Was it easy to get?
**Grand Tour Question 6: How did physical changes impact on your ability to participate in activities that positively contributed to your wellbeing?**
**Draft set:**
• What resources did you have access to that helped you physically?
• What resources did you find on your own that your doctor did not tell you about, if any?
• What resources would have made the physical challenges you faced easier?
• How did physical changes impact you or your loved one's ability to engage in activities that brought them happiness or activities you or your loved one engaged in before their stroke?
• If you or your loved one were working prior to the stroke, how did it impact their job?
**<u>Emotional Wellbeing:</u>**
**Session Opening Questions**
• What does emotional wellbeing mean to you? And how did it change after a stroke?
**1) Grand Tour Questions:**
• How does spirituality impact health and recovery?
• How does your social community impact your health and recovery?
**Clarification Questions- Emotional Wellbeing**
• How does emotional wellbeing change after a stroke?

o Did you notice a change in your emotions after stroke?
o Did anyone talk to you about emotional wellbeing after your stroke? A doctor? Therapist?
• How did your social network or community impact your emotional wellbeing?
o What were the parts of your community/social network **that helped** wellbeing?
o What were the parts of your community/social network that made wellbeing **more difficult** to achieve?
• How did your neighborhood environment change after a stroke? – physical environment that you live in
o Did you feel like the environment you lived in had an impact on your emotional wellbeing?
o What parts of your neighborhood helped wellbeing?
o What parts of your neighborhood made achieving wellbeing more difficult?
o Did you wish you had other resources in your environment?
• A lot of you have mentioned counseling, how did that impact your emotional wellbeing?
**Clarification Questions- Spiritual Wellbeing**
• How does spirituality impact health?
• How does spirituality impact wellbeing?
• How does spiritual wellbeing change after a stroke?
o What does spiritual wellbeing mean? And how did it change after a stroke
o Did you notice a change in your spirituality after stroke?
o Did you ever meet with a faith-leader in the hospital or afterwards to talk about your stroke?
• How did your social network or community impact your spiritual wellbeing?
o What were the parts of your community/social network **that helped** wellbeing?
o What were the parts of your community/social network that made wellbeing **more difficult** to achieve?
• How did your neighborhood environment change after a stroke? – physical environment that you live in
o Did you feel like the environment you lived in had an impact on your spiritual wellbeing?
o Were you able to return to your place of worship after your stroke?
o What parts of your neighborhood helped wellbeing?
o What parts of your neighborhood made achieving wellbeing more difficult?
Social Community
• When you refer to your social community, are you thinking primarily of family, friends, or a broader network?
• Could you specify the kinds of support or influence you find most significant within your social community?

© 2025 Choksi D et al. *JAMA Network Open.*
