# Supplementary material for: Coherence of Stroke Survivors’ Lived Experiences and the Stroke-Specific Quality of Life Scale
Source: JAMA Netw Open. 2025 Oct 17;8(10):e2537951. doi: 10.1001/jamanetworkopen.2025.37951 (PMC12534846; doi:10.1001/jamanetworkopen.2025.37951)
Supplement: Supplement 2. — Data Sharing Statement [file jamanetwopen-e2537951-s002.pdf]

Data Sharing Statement

Choksi. Coherence of Stroke Survivors' Lived Experiences and the Stroke-Specific Quality of Life Scale. *JAMA Netw Open*. Published October 17, 2025.
doi:10.1001/jamanetworkopen.2025.37951

Data
Data available: Yes
Data types: Deidentified participant data
How to access data: How to access the data: Please contact the study's principal investigator Dr. Nirupama Yechoor at nyechoor@mgh.harvard.edu to request data access.
When available: With publication

Supporting Documents
Document types: None

Additional Information
Who can access the data: Due to the potential possibility of identifiable information being present even after rigorous de-identification of transcripts, the full qualitative dataset is not openly shared. The research team welcomes data-sharing inquiries and is willing to provide de-identified transcripts on a case-by-case basis under a data-sharing agreement and appropriate IRB approval.
Types of analyses: Qualitative Analysis
Mechanisms of data availability: With signed data use agreement on a case-by-case basis
Any additional restrictions: None
